# Ginsenoside Rb1 Improves Metabolic Disorder in High-Fat Diet-Induced Obese Mice Associated With Modulation of Gut Microbiota

**DOI:** 10.3389/fmicb.2022.826487

**Published:** 2022-04-19

**Authors:** Hong Zou, Man Zhang, Xiaoting Zhu, Liyan Zhu, Shuo Chen, Mingjing Luo, Qinglian Xie, Yue Chen, Kangxi Zhang, Qingyun Bu, Yuchen Wei, Tao Ye, Qiang Li, Xing Yan, Zhihua Zhou, Chen Yang, Yu Li, Haokui Zhou, Chenhong Zhang, Xiaoyan You, Guangyong Zheng, Guoping Zhao

**Affiliations:** ^1^State Key Laboratory of Genetic Engineering, Department of Microbiology and Immunology, School of Life Sciences, Fudan University, Shanghai, China; ^2^Engineering Laboratory for Nutrition, Shanghai Institute of Nutrition and Health, Chinese Academy of Sciences, Shanghai, China; ^3^Master Lab for Innovative Application of Nature Products, National Center of Technology Innovation for Synthetic Biology, Tianjin Institute of Industrial Biotechnology, Chinese Academy of Sciences, Tianjin, China; ^4^Zhejiang Hongguan Bio-Pharma Co., Ltd., Jiaxing, China; ^5^CAS Key Laboratory of Quantitative Engineering Biology, Shenzhen Institute of Synthetic Biology, Shenzhen Institute of Advanced Technology, Chinese Academy of Sciences, Shenzhen, China; ^6^Department of Microbiology, The Chinese University of Hong Kong, Hong Kong, China; ^7^Suzhou BiomeMatch Therapeutics Co., Ltd., Shanghai, China; ^8^CAS-Key Laboratory of Synthetic Biology, CAS Center for Excellence in Molecular Plant Sciences, Shanghai Institute of Plant Physiology and Ecology, Chinese Academy of Sciences, Shanghai, China; ^9^State Key Laboratory of Microbial Metabolism, School of Life Sciences and Biotechnology, Shanghai Jiao Tong University, Shanghai, China; ^10^College of Food and Bioengineering, Henan University of Science and Technology, Luoyang, China; ^11^Bio-Med Big Data Center, Shanghai Institute of Nutrition and Health, Chinese Academy of Sciences, Shanghai, China

**Keywords:** ginsenoside Rb1, metabolic disorder, gut microbiota, long-chain fatty acids, free fatty acid receptor, lipidomics, fecal metabolome

## Abstract

Gut microbiota plays an important role in metabolic homeostasis. Previous studies demonstrated that ginsenoside Rb1 might improve obesity-induced metabolic disorders through regulating glucose and lipid metabolism in the liver and adipose tissues. Due to low bioavailability and enrichment in the intestinal tract of Rb1, we hypothesized that modulation of the gut microbiota might account for its pharmacological effects as well. Here, we show that oral administration of Rb1 significantly decreased serum LDL-c, TG, insulin, and insulin resistance index (HOMA-IR) in mice with a high-fat diet (HFD). Dynamic profiling of the gut microbiota showed that this metabolic improvement was accompanied by restoring of relative abundance of some key bacterial genera. In addition, the free fatty acids profiles in feces were significantly different between the HFD-fed mice with or without Rb1. The content of eight long-chain fatty acids (LCFAs) was significantly increased in mice with Rb1, which was positively correlated with the increase of *Akkermansia* and *Parasuttereller*, and negatively correlated with the decrease of *Oscillibacter* and *Intestinimonas*. Among these eight increased LCFAs, eicosapentaenoic acid (EPA), octadecenoic acids, and myristic acid were positively correlated with metabolic improvement. Furthermore, the colonic expression of the *free fatty acid receptors 4* (*Ffar4*) gene was significantly upregulated after Rb1 treatment, in response to a notable increase of LCFA in feces. These findings suggested that Rb1 likely modulated the gut microbiota and intestinal free fatty acids profiles, which should be beneficial for the improvement of metabolic disorders in HFD-fed mice. This study provides a novel mechanism of Rb1 for the treatment of metabolic disorders induced by obesity, which may provide a therapeutic avenue for the development of new nutraceutical-based remedies for treating metabolic diseases, such as hyperlipidemia, insulin resistance, and type 2 diabetes.

## Introduction

The prevalence of obesity and related comorbidities continues to rise worldwide. The prevention and treatment of obesity and its metabolic complications remain a major public health concern.

Gut microbiota plays an important role in the development of obesity with related hyperlipidemia and diabetic symptoms (Festi et al., 2014). Intestinal microbes affect nutrient acquisition and energy regulation of the host (Kimura et al., 2013). Gut microbiota dysbiosis damages the intestinal epithelial barrier and intestinal mucosal immunity, which leads to insulin resistance and inflammation (Paone and Cani, 2020). Thus, modulating the gut microbiota may be an effective strategy for the treatment of obesity and related metabolic disorders.

Metabolomic analysis can deepen the understanding of host-microbe interactions (Heinken and Thiele, 2015). The fecal metabolome investigates the outcomes of the metabolic interactions among diet, gut microbiota and host through analyzing the remaining unabsorbed metabolites (Matysik et al., 2016). Lipidomics study may be useful in understanding the contribution of fatty acids and lipids profile toward maintaining or disturbing the metabolic homeostasis, particularly indicated by the readouts of insulin resistance (IR) and obesity (Shetty and Kumari, 2021).

*Panax ginseng* and *Panax notoginseng* are widely used for 1000 of years in Asia due to their effectiveness in strengthening health and recovering from deficiency of “vital energy (*QI*).” Ginsenosides are the main bioactive components. Ginsenoside Rb1 (Rb1) is the most abundant and thought to be an important active factor in protopanaxadiol ginsenosides. Previous studies have shown that ginsenoside Rb1 exerts significant anti-obesity and anti-diabetic effects (Zhou et al., 2019). Rb1 can decrease body weight, increase insulin sensitivity, suppress liver fat accumulation, regulate adipocyte function and improve glucose tolerance in obese mice and rats (Xiong et al., 2010; Shen et al., 2013, 2015; Lin et al., 2014; Yu et al., 2015; Song et al., 2017; Lim et al., 2019). Rb1 exerts multi-target effects and its molecular mechanism mainly involves in activating AMP-activated protein kinase (Shen et al., 2013; Zhao et al., 2019) and regulating PPARγ signaling (Mu et al., 2015; Song et al., 2020). However, these pharmacological effects were observed in the treatment *via* intraperitoneal injection of Rb1, and the molecular targets analyzed were mainly in the liver, skeletal muscle and adipose tissues. It is well known that most herbal medicine, including ginsenosides, are orally administrated. However, the oral bioavailability of Rb1 is relatively low, around 0.1–4.35%(Odani et al., 1983; Xu et al., 2003; Liu et al., 2015). After being orally administrated, the levels of Rb1 in the blood and target tissues are far below the effective concentrations used in intraperitoneal administration studies. So, the AMPK and PPARγ pathways may not actually be followed *in vivo* by Rb1 via oral administration. Therefore, the current mechanistic hypotheses of Rb1 are insufficient to explain the results of *in vivo* animal studies which are orally given with Rb1.

Since poor bioavailability, Rb1 concentration is low in the plasma but is very high in the gut (especially in the large intestine), which provides the possibility of interaction between Rb1 and the gut microbes. In addition, it has been reported that after Rb1 was orally administrated, intestinal microbes were involved in the transformation of Rb1. β- D-glucosidase produced by gut microbes converts Rb1 into other saponins (Akao et al., 1998a). Germ-free rats (Akao et al., 1998b) and factors affecting gut microbes such as antibiotics (Xu et al., 2014), stress (Kang et al., 2016) etc., affect Rb1 degradation into other saponins and also Rb1 pharmacokinetics. Therefore, we hypothesize that the gut microbiota might be an alternative therapeutic target for Rb1.

Recent studies have reported that some saponins treated by oral administration could regulate gut microbiota and metabolites profiles to improve physiological indices. For instance, *Panax notoginseng* saponins modulate the gut microbiota accompanied by increasing fecal fatty acids, which promotes adipose thermogenesis in diet-induced obese mice (Xu et al., 2020). Ginseng extract can increase *Enterococcus faecalis* that can produce myristoleic acid, which leads to reducing adiposity (Quan et al., 2020). However, there is limited data about the influences of pure Rb1 on gut microbiota and metabolites. In this study, in the process of evaluating the anti-obesity effects of Rb1 in HFD-fed mice, we studied the alteration of gut microbiota and profiles of fecal metabolites in response to Rb1 treatment. Through correlation analysis, we explored the multiscale mechanisms that might account for the therapeutic effect of Rb1 against obesity.

## Materials and Methods

### Chemicals

Ginsenoside Rb1 was purchased from the FEIYUBIO (Nantong, China). Rb1 was dissolved in saline and gavaged to mice or rats.

### Animals and Experimental Design

C57BL/6J mice (Shanghai Laboratory Animal Co.) and SD rats (Beijing Vital River Laboratory Animal Technology Co.) were raised under a 12:12 h light/dark cycle in a temperature and humidity-controlled room.

#### High-Fat Diet-Induced Obese Mouse Model

Mice were divided into three groups: (1) ND: normal diet treated with saline (*n* = 5), (2) HFD: high-fat diet treated with saline (*n* = 5), (3) Rb1: high-fat diet treated with Rb1 at a dose of 120 mg/kg (*n* = 7). Male mice of 7 weeks of age were provided with a normal chow diet or high-fat diet (D12492: 60% fat, 20% carbohydRate, 20% protein, Research Diets, Inc., NY, United States) for 12 weeks. Then mice in the ND group and the HFD group were gavaged with saline and mice in the Rb1 group were gavaged with Rb1 for 28 days. Blood samples were collected after Rb1 treatment for 24 days after mice fasting overnight. Fecal samples of mice were collected before Rb1 treatment (day 0) and after Rb1 treatment for 9, 20, 24, and 27 days (day 9, day 20, day 24, and day 27). At the end of the trial, mice fasted overnight and proximal colon, liver and epididymal fat tissues were collected. Samples of serum, feces, and tissues were stored at –80°C.

#### Diet-Induced Hyperlipemia Rat Model

Male SD rats were divided into three groups (8 rats per group): (1) a normal diet treated with saline (ND), (2) a high-fat diet treated with saline (HFD), (3) a high-fat diet treated with Rb1 (Rb1, 60 mg/kg). Firstly, rats were fed with the normal diets or normal diets added with 20.0% sucrose, 15% lard, 1.2% cholesterol, and 0.2% sodium cholate for 2 weeks. Blood was collected and group division based on the level of serum total cholesterol (TC). Then rats were gavaged with Rb1. After four weeks of treatment, blood was obtained and stored at –80°C. The liver and epididymal fat were collected for histopathology analysis.

### Measurement of Blood Chemistry and Hepatic Lipid Contents

Serum triglycerides (TG), total cholesterol (TC), low-density lipoprotein cholesterol (LDL-c), and glucose were measured using commercial detection kits (NJJCBIO Co., Ltd., Nanjing, China). Glycosylated hemoglobin (GHb) was tested using a commercial kit (Jianglai Biotechnology Co., Ltd., Shanghai). Insulin was determined using a commercial ELISA kit (Mercodia). Insulin resistance was assessed using the index of HOMA-IR: fasting blood glucose (mmol/L) × fasting blood insulin (mU/L)/22.5. Hepatic lipids from the mice liver homogenate were extracted and hepatic TG and TC were determined using assay kits (NJJCBIO Co., Ltd., Nanjing, China).

### Intraperitoneal Glucose Tolerance Test

Intraperitoneal glucose tolerance test was performed after Rb1 treatment for 16 days. After fasting for 6h, mice were given glucose (2 g/kg body weight) intraperitoneally. Tail blood was used to inspect glucose levels at 0, 15, 30, 60, and 120 min after glucose injection.

### Analysis of Histopathology

The liver and epididymis fat tissues of rats were fixed with formalin and embedded in paraffin. 4 μm sections were stained with hematoxylin and eosin (H&E). For hepatic fat accumulation analysis, livers were embedded in frozen sections and 10 μm sections were stained with oil red O (ORO).

### RNA Isolation and Real-Time Quantitative PCR Analysis

Total RNA was extracted from the colon tissues with RNeasy Plus Universal Mini Kit (QIAGEN). Then cDNA was prepared with PrimeScript RT reagent Kit (TAKARA). Quantitative real-time PCR (qRT-PCR) was performed with SYBR Premix Ex TaqTM (TAKARA) on the Step-One-Plus Real-Time PCR Systems (Applied Biosystem) by using Gapdh as the internal control. Primers that were used in qRT-PCR were shown in Supplementary Table 1.

### Gut Microbiota Detection and Microbial Function Prediction

Fecal samples of mice collected at days 0, 9, 20, and 27 were used for gut microbiota profiling. Genomic DNA was extracted from feces with a QIAamp DNA stool mini kit (QIAGEN, Germany). Subsequently, the V3–V4 region of the 16S rRNA gene was amplified by PCR and then amplicons were purified and sequenced on a MiSeq system.

Sequencing reads were analyzed using the QIIME II software (Bolyen et al., 2019). In practice, chimeric sequences and low-quality sequencing reads were filtered by the DADA2 method included in the qiime2 software, which was an ASV-based community comparison method for 16S amplicon data analysis (Callahan et al., 2016). After that, high-quality reads were kept to build a feature table, which contained counts (frequencies) of each unique sequence in each sample. Then taxonomic annotation analysis based on the feature table was carried out to explore the microbial profile of samples. Subsequently, alpha and beta diversity analyses were conducted. In detail, the alpha diversity of each group was calculated by the Shannon index and Pielou evenness index; while the beta diversity was calculated with the principal coordinate analysis (PCoA) method based on the unweighted UniFrac distance matrices. Adonis (permutational multivariate analysis of variance using distance matrices) was used to compare the significant difference between groups. To identify microbial markers between groups, the approach of linear discriminant analysis effect size (LEfSe) was applied and the threshold of linear discriminant analysis (LDA) score was set to 3.0. Finally, based on the feature table, the tax4fun software (Aßhauer et al., 2015) was adopted to infer involved signal pathways of microbes so as to predict the functional profiles of the microbial communities.

### Fecal Metabolite Analysis

Fecal samples of mice collected on day 24 after Rb1 treatment were used for metabolite analysis.

#### Metabolite Extractions

To extract metabolites from fecal samples, 800 μL of cold methanol/acetonitrile/water (2:2:1, vol/vol/vol) extraction solvent was added to 30–80 mg feces, and vortexed adequately. Stock solutions of stable-isotope internal standards were added to the extraction solvent simultaneously. Then samples were under vigorous shaking (2 min) and followed by incubation (20 min, 4°C). After centrifugation (14,000 g, 20 min, 4°C), the supernatant flowed through a 96-well protein precipitation plate, the elution was vacuum-dried at 4°C. Then the samples were re-dissolved in 100 μL acetonitrile/water (1:1:1, vol/vol/vol) solvent and transferred to LC vials.

#### LC-MS/MS Analysis

Analyses were performed using a UHPLC (1290 Infinity LC, Agilent Technologies) coupled to a 6500 QTRAP MS (AB Sciex). The analytes were separated on UPLC BEH C18 columns (2.1 × 100 mm, 1.7 μm; Waters). The column temperature was set at 40°C, and the injection volume was 2 μL. The mobile phase A was 50 mM ammonium formate and 0.4% formic acid in the water, and B was methanol. A gradient (5% B at 0 min, 60% B at 5 min, 100% B at 11–13 min, 5% B at 13.1–16 min) was then initiated at a flow rate of 400 μL/min. The sample was placed at 4°C during the whole analysis process. 6500 QTRAP was performed in positive and negative switch mode. The ESI positive source conditions were set as follows: source temperature: 550°C; ion Source Gas1: 55; Ion Source Gas2: 55; Curtain gas: 40; ion Sapary Voltage Floating: + 4500. The ESI negative source conditions were set as follows: source temperature: 550°C; ion Source Gas1: 55; Ion Source Gas2: 55; Curtain gas: 40; ion Sapary Voltage Floating: –4500 V. MRM method was used for mass spectrometry quantitative data acquisition. A pooled sample was set in the sample queue as quality control (QC) to evaluate the stability and repeatability of the system.

#### Data Processing

The Analyst software was used for quantitative data processing. The QCs were processed together with the biological samples. Peak values of each metabolite were normalized by the weight of the feces and peaks of internal standards.

### Statistical Analysis

The student’s *t*-test was performed to compare data between two groups and a one-way analysis of variance followed by Tukey’s *post hoc* test was applied to compare data among more than two groups. To compare values among three groups of different time points, a two-way analysis of variance followed by Tukey’s multiple comparison test was performed. To evaluate the correlation between physiology phenotypes, gut microbes, or fecal metabolites, *Spearman*’s correlation analysis was used. A *P*-value < 0.05 was considered statistically significant. Data were expressed as means ± SEM (standard errors of the means).

## Results

### Orally Administrated Ginsenoside Rb1 Improves Insulin Resistance and Lipid Metabolism in High-Fat Diet-Fed Rodents

**FIGURE 1:**
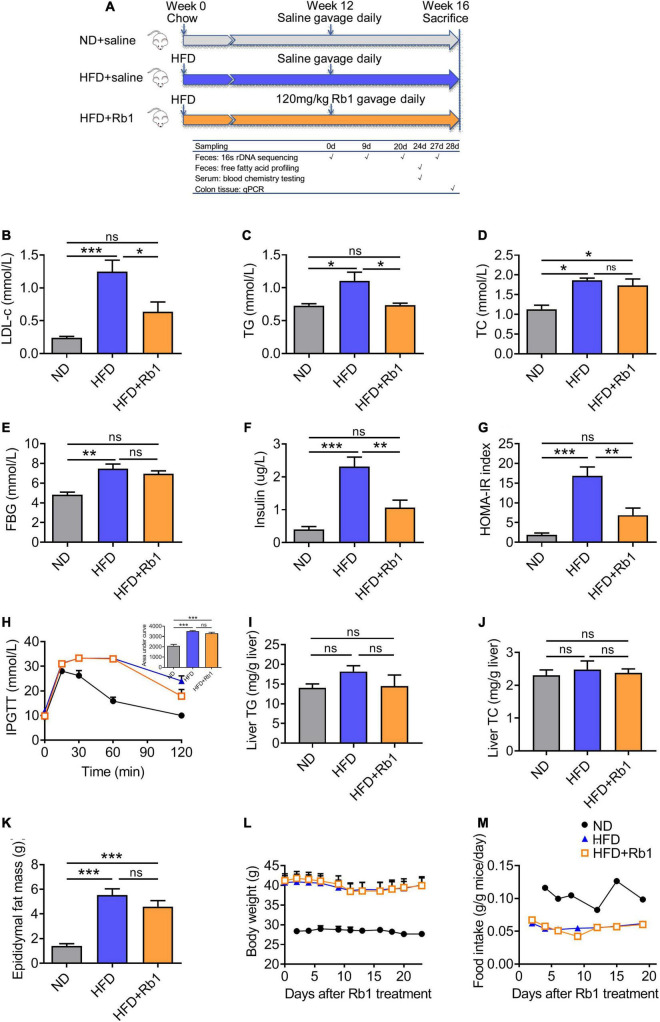
Rb1 treatment improved lipid metabolism and insulin resistance in high-fat diet (HFD)-fed mice. **(A)** Mice were fed with a high-fat diet for 12 weeks and then subjected to Rb1 treatment (120 mg/kg) by gavage for 4 weeks. Blood samples of day 24 were used to test serum metabolic parameters. Tissues were collected on day 28. **(B)** Low-density lipoprotein cholesterol (LDL-c), **(C)** triglyceride (TG), **(D)** total cholesterol (TC), **(E)** fasting blood glucose (FBG), **(F)** fasting insulin was tested in serum and **(G)** homeostasis model assessment of insulin resistance (HOMA-IR) index was assessed. **(H)** An Intraperitoneal glucose tolerance test was performed on day 16. **(I)** Liver TG, **(J)** liver TC, and **(K)** epididymal fat were assessed. **(L)** Body weight and **(M)** food intake data were recorded twice a week. All data were presented as the mean ± SEM (*n* = 5–7). A one-way analysis of variance followed by Tukey’s *post hoc* test was applied to compare data among groups. *P < 0.05; **P < 0.01; ***P < 0.001. ns, not significant.

A high-fat diet-fed obese model was established by feeding C57BL/6J male mice with a high-fat diet for 12 weeks. Therapeutics via daily oral administration of Rb1 was performed in the following 4 weeks (Figure 1A). Compared to the HFD group, Rb1 treatment significantly lowered the serum LDL-c (*P* < 0.05, Figure 1B) and TG (*P* < 0.05, Figure 1C), with no effect on the serum TC, body weight, and food intake (Figures 1D,L,M). On the other hand, Rb1 unaffected either fasting blood glucose (Figure 1E) or glucose tolerance (Figure 1H), but significantly lowered the fasting blood insulin (*P* < 0.01, Figure 1F) and the HOMA-IR index (*P* < 0.01, Figure 1G). In addition, compared to the HFD group, Rb1 lowed liver TG and epididymal fat mass for a trend but had no effect on liver TC (Figures 1I,J,K).

**FIGURE 2:**
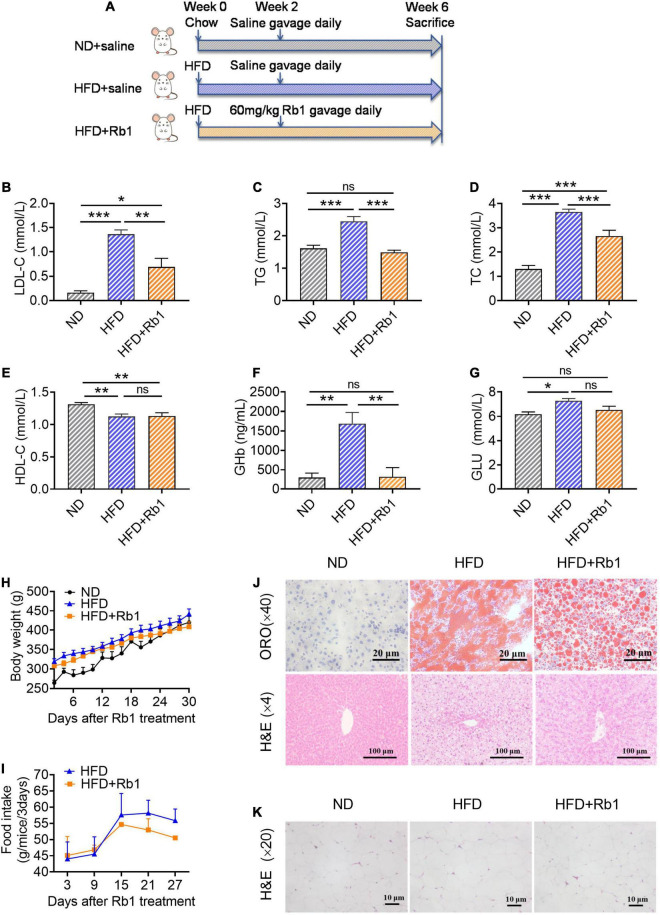
Rb1 treatment improved lipid metabolism in HFD-fed rats. **(A)** Rats were provided with a high-fat diet for 2 weeks and then subjected to treatment with Rb1 (60 mg/kg) by gavage for 4 weeks. Blood samples of day 28 were used to test serum metabolic parameters, including **(B)** low-density lipoprotein cholesterol (LDL-c), **(C)** triglyceride (TG), **(D)** total cholesterol (TC), **(E)** high-density lipoprotein cholesterol (HDL-c), **(F)** glycosylated hemoglobin (GHb), and **(G)** blood glucose (Glu). **(H)** Body weight and **(I)** food intake were also recorded. **(J)** Oil red O (ORO) staining and hematoxylin and eosin (H&E) staining of liver tissues. **(K)** H&E staining of epididymal fat tissues. All data were expressed as the mean ± SEM (*n* = 8). Significant difference among groups were evaluated using one-way analysis of variance followed by Duncan’s *post hoc* test. *P < 0.05; **P < 0.01; ***P < 0.001. ns, not significant.

These results were reproduced in the diet-induced hyperlipemia rat model (Figure 2A). As shown in Figure 2, compared to the HFD group, Rb1 could significantly decrease the levels of LDL-c (*P* < 0.001, Figure 2B), TG (*P* < 0.01, Figure 2C) and TC (*P* < 0.05, Figure 2D), and did not affect HDL-c, body weight and food intake (Figures 2E,H,I). Meanwhile, the GHb (*P* < 0.01, Figure 2F) detected in the Rb1 group was much lower compared to that of the HFD group although blood glucose showed only a trend of decline (Figure 2G). Rb1 also reduced the accumulation of lipid droplets in the liver tissues of rats observed by oil red O staining and H&E staining (Figure 2J). The results of H&E staining of epididymal fat tissues showed that the adipocytes in the Rb1 group were significantly smaller than those in the HFD group (Figure 2K).

Taken together, Rb1 treatment has consistent results in mice and rats and thus strongly suggests that Rb1 treatment improves the lipid metabolism and insulin resistance in HFD-fed rodents. For further exploration of the possible mechanisms of Rb1 function, only mice samples were used for evaluation.

### Rb1 Treatment Partly Recovers the Gut Microbiota Destructed by High-Fat Diet in Mice

To explore whether gut microbiota plays a significant role in Rb1 treatment in HFD-fed rodents, mouse fecal samples collected at day 0, day 9, day 20, and day 27 were used to analyze the dynamic change of gut microbiota. The bacterial 16S rRNA (V3-V4 region) gene was sequenced for these fecal samples. After removing low-quality sequencing reads (Methods), a total of 55M raw reads, and an average of 0.8M reads per sample were obtained. Subsequently, feature table construction and taxonomic annotation were conducted to generate the microbial profiles for evaluating the alteration of gut microbiota for different groups of mice.

**FIGURE 3:**
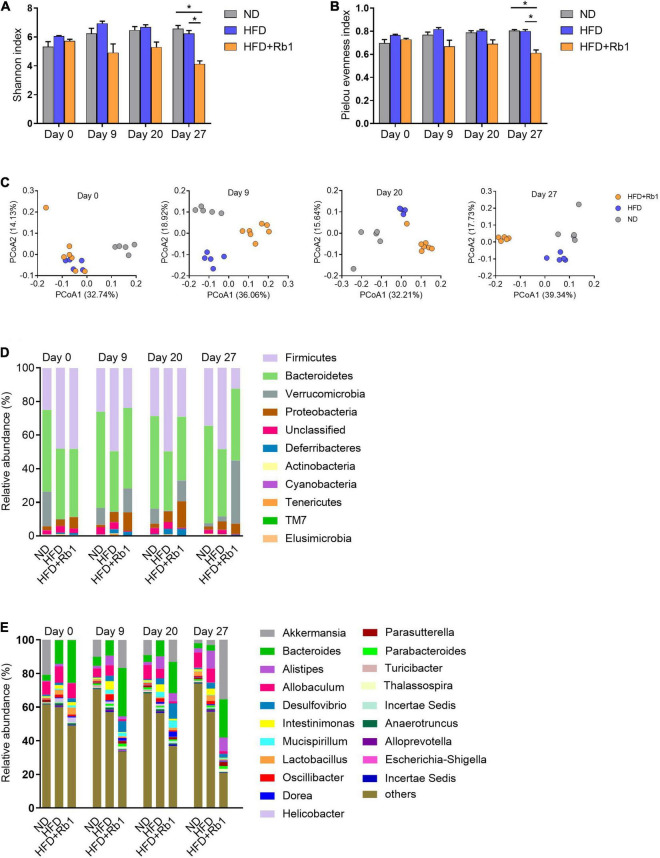
Rb1 treatment altered the composition of gut microbiota in HFD-fed mice. Fecal samples of mice were collected before Rb1 treatment (day 0) and after Rb1 treatment (day 9, day 20, and day 27). Bacterial 16S rRNA (V3-V4 region) sequencing was conducted for these fecal samples. The alpha diversity was estimated by the **(A)** Shannon Index and **(B)** Pielou evenness index. The beta diversity was assessed by **(C)** principal coordinates analysis (PCoA). Relative abundance of taxa **(D)** at the phylum level and **(E)** at the genus level is shown. All data were expressed as the mean ± SEM (*n* = 5–7). Significant difference among groups were evaluated using two-way analysis of variance followed by Tukey’s multiple comparison test. *P < 0.05; ** *P* < 0.01; ***P < 0.001.

The alpha diversity was estimated by the Shannon index and Pielou evenness index. As shown in Figures 3A,B, both Shannon index and Pielou evenness index of the Rb1 group began to decrease from day 9 and were significantly lower than those of the HFD group on day 27 (*P* < 0.05), showing an obvious temporal effect with Rb1 treatment. And this result indicated that Rb1 might reduce the diversity of the gut microbes of HFD-fed mice. The beta diversity was estimated by PCoA based on the unweighted UniFrac distance matrices. For mice in the Rb1 group, they were classified into an independent cluster compared to those of the HFD group on day 9 (Adonis, *P* < 0.01), day 20 (Adonis, *P* < 0.01), and day 27 (Adonis, *P* < 0.05), whereas mice from two HFD-fed groups were classified into the same cluster on day 0 (Adonis, *P* > 0.05) (Figure 3C). The results indicated that Rb1 treatment might alter the gut microbial composition of HFD-fed mice.

Additionally, the gut microbial composition was classified and exhibited at the phylum level (Figure 3D) and the genus level (Figure 3E). As shown in Supplementary Figures 1A-D, the relative abundance of Firmicutes was significantly increased by HFD and decreased by Rb1 treatment, meanwhile, HFD increased the ratio of Firmicutes to Bacteroidetes, whereas Rb1 reversed. Notably, compared with the HFD group, Rb1 treatment significantly increased the relative abundance of *Akkermansia* which belongs to Verrucomicrobia (Figures 3D, E).

**FIGURE 4:**
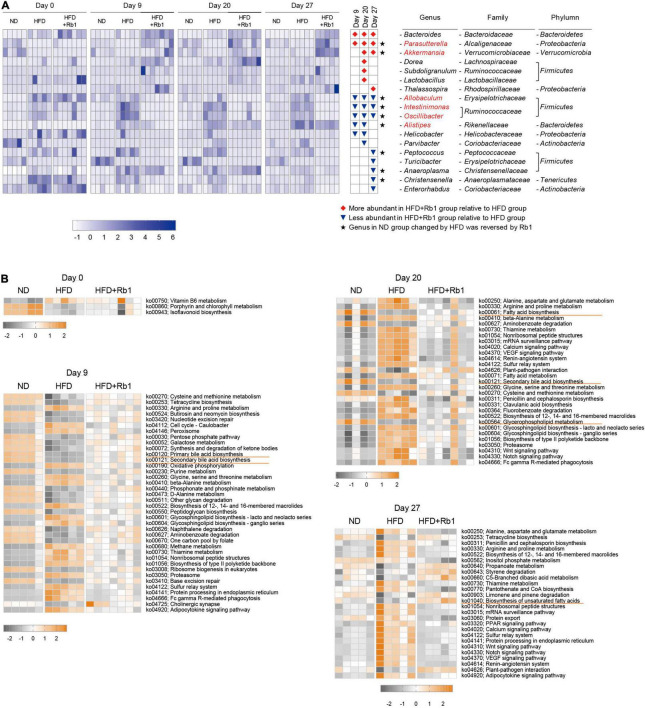
Key bacterial alterations in response to Rb1 treatment in HFD-fed mice and functional pathway prediction. Key genera significantly altered by Rb1 treatment were selected by Linear discriminate analysis effect size (LEfSe) analysis. Functional pathway prediction was performed by the tax4fun software. **(A)** Heatmap presents the relative abundance of key genera significantly altered by Rb1 treatment (LDA scores > 3)**. (B)** Heatmap presents the abundance of differential pathways in day 0, day 9, day 20, and day 27. Pathways with significant differences between Rb1 and HFD are shown in the top 20 (pathways with the same *P-*value are shown all). The abundance of pathways was normalized by Z-score method. Kruskal-Wallis rank-sum test was used to calculate the significant difference between groups (*P* < 0.05).

To identify the specific intestinal bacterial markers responding to Rb1 treatment, linear discriminate analysis effect size (LEfSe) analysis was conducted (Supplementary Figure 2). As shown in Figure 4A, compared to the HFD group, a total of 18 genera were significantly altered in abundance by Rb1 treatment, with seven genera increased and eleven genera decreased, at each time point respectively. Among them, eight genera were significantly altered at two or three-time points, indicating the solid and persistent response of these genera to Rb1 treatment. Three of them were enriched, including *Bacteroides*, *Parasutterella*, and *Akkermansia* (highlighted with red diamonds in Figure 4A) and the remaining five of them were reduced, including *Allobaculum*, *Intestinimonas*, *Oscillibacter*, *Alistipes, and Helicobacter* (highlighted with blue triangles in Figure 4A). Notably, 6 genera were recovered by Rb1 treatment which were previously altered by the high-fat diet, including *Parasutterella*, *Akkermansia*, *Allobaculum*, *Intestinimonas*, *Oscillibacter*, and *Alistipe*s (highlighted in red text in Figure 4A).

Furthermore, microbial functions were predicted using tax4fun software to find out differential functional pathways among the three groups. Figure 4B shows the main significantly differential pathways in response to Rb1 treatment in different time points. Compared with the HFD group, a higher abundance of gut microbiota in the Rb1 groups was associated with pathways involved in lipids metabolism, including fatty acid biosynthesis (day 20), biosynthesis of unsaturated fatty acids (day 27), primary bile acid biosynthesis (day 9) and secondary bile acid biosynthesis (day 9 and 20). On the contrary, compared with the ND group, a lower abundance of gut microbiota in the HFD group was associated with these pathways.

These results indicate that oral administration of Rb1 modulates the gut microbiota, and partially reverses the gut dysbiosis in HFD-Fed mice. Microbial functional pathway prediction indicates pathways associated with lipids metabolism.

### Rb1 Treatment Increases Long-Chain Fatty Acids in the Feces of High-Fat Diet-Fed Mice

**FIGURE 5:**
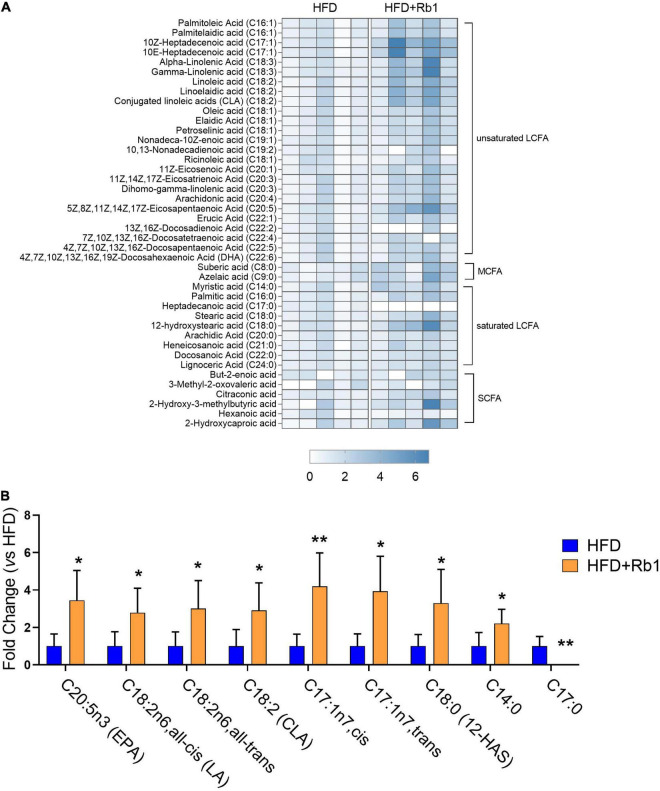
Rb1 modified fecal free fatty acids profiles in HFD-fed mice. The content of free fatty acids in fecal samples collected after Rb1 treatment for 24 days was determined by LC-MS/MS. **(A)** Heatmap of fecal free fatty acids in mice of the HFD and Rb1 group is shown. **(B)** Fold change of differential fecal free fatty acids of the Rb1 group compared to the HFD group is displayed. All data were expressed as the mean ± SEM (*n* = 5). *P*-values were evaluated using a *t*-test analysis. *P < 0.05; **P < 0.01. LCFA, long-chain fatty acid, MCFA: medium-chain fatty acid; SCFA, short-chain fatty acid.

Considering that gut microbiota of the Rb1 group was predicted to play a role in lipid metabolism, especially in fatty acid metabolism, we further measured the content of free fatty acids in fecal samples which were collected after Rb1 treatment for 24 days. As shown in Figure 5A, a total of 42 free fatty acids was detected, including six short-chain fatty acids, two medium-chain fatty acids, nine long-chain saturated fatty acids, and 25 long-chain unsaturated fatty acids.

As shown in Figure 5B, compared to the HFD group, a total of nine free fatty acids were significantly changed in the Rb1 group (Figure 5B), with eight increased and one decreased. Among eight increased fatty acids, six were long-chain unsaturated fatty acids. Compared to the HFD group, eicosapentaenoic acid (EPA, C20:5n3, *P* < 0.05) was significantly increased by 3.5 times in the Rb1 group. Three octadecenoic acids, including linoleic acid (C18:2n6, all-cis, *P* < 0.05), linoelaidic acid (C18:2n6, all-trans, *P* < 0.05), and conjugated linoleic acids (CLA, C18:2, *P* < 0.05), were significantly increased by 2.8, 3.0 and 2.9 times respectively in the Rb1 group. Additionally, 10Z-heptadecenoic acid (C17:1n-7, cis, *P* < 0.01) and 10E-heptadecenoic acid (C17:1n-7, trans, *P* < 0.05) were increased by 4.2 and 3.9 times respectively, while heptadecanoic acid (C17:0, *P* < 0.01) was decreased (not detected in the Rb1 group). Furthermore, two long-chain saturated fatty acids, including 12-hydroxystearic acid (12-HAS, C18:0, *P* < 0.05) and myristic acid (C14:0, *P* < 0.05) were significantly increased by 3.3 and 2.2 times respectively.

These results suggest that Rb1 treatment increases the content of a group of free fatty acids in feces, especially long-chain unsaturated fatty acids including EPA and octadecenoic acids.

### Rb1 Treatment Adjusts Expression of Free Fatty Acid Receptor-Related Genes in the Colon of High-Fat Diet-Fed Mice

**FIGURE 6:**
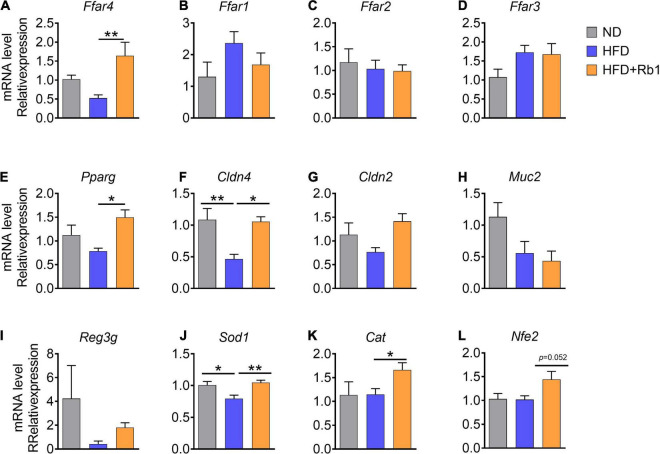
Rb1 treatment adjusted colonic gene expression associated with free fatty acids receptors and the intestinal barrier function in HFD-fed mice. Relative mRNA expression in colon was determined by real-time PCR. **(A–E)** Free fatty acids receptor-related genes, **(F–I)** intestinal barrier function-related genes, **(J–L)** oxidate stress-related genes are shown. Relative expression levels were normalized to those of GAPDH. All data were expressed as the mean ± SEM (*n* = 5–7). *P-*values were determined using a *t*-test analysis. *P < 0.05; **P < 0.01.

Increasing evidence indicated that free fatty acids are natural ligands for a group of free fatty acid receptors (FFARs). To date, four FFARs have been identified (Kimura et al., 2020). *Ffar2* and *Ffar3* are activated by SCFAs (Brown et al., 2003), *Ffar1* is activated by MCFAs (Briscoe et al., 2003), while *Ffar4* is activated by LCFAs (especially unsaturated LCFAs) (Hirasawa et al., 2005). In this study, we tested FFARs gene expression in the colon to find out whether their expression was associated with the increase of free fatty acids induced by Rb1 treatment. Our results showed that the expression of *Ffar4* was significantly increased in the Rb1 group (*P* < 0.01), while it was decreased in the HFD group (Figure 6A). And other fatty acid receptors including *Ffar1* (Figure 6B), *Ffar2* (Figure 6C), and *Ffar3* (Figure 6D) were unaffected with Rb1 treatment.

Additionally, *Pparg* which can be activated by unsaturated LCFAs and abundant in the colon (Su et al., 2007) was also significantly upregulated by Rb1 (*P* < 0.05, Figure 6E).

Previous reports show that diets and gut microbes may affect intestinal barrier function (Moreira et al., 2012; Rohr et al., 2020). Tight junction protein and antimicrobial peptides in the intestine are contributing to the gut barrier function (Everard et al., 2014; Zihni et al., 2016). A high-fat diet can induce oxidative stress in the intestine which triggers gut barrier injury and endotoxemia (Moreira et al., 2012; Rohr et al., 2020). Here, we further assessed the gene expression of several marker genes associated with intestinal barrier function in the colon. Claudin consists of the main chain of the tight junction complex (Venugopal et al., 2019). We found that the expression of *Cldn4* (encoding claudin 4) and *Cldn2* (encoding claudin 2) were higher in mice treated with Rb1 when compared to untreated HFD-fed mice (*Cldn4*, *P* < 0.05, Figures 6F,G), while *Muc2* gene expression was unaffected (Figure 6H). Additionally, higher expression of antimicrobial peptides gene *Reg3g* was observed in the Rb1 group than that in the HFD group for a trend (Figure 6I). Our results showed that Rb1 significantly increased the expression of oxidative-stress-related genes in the colon, including *Sod1*(*P* < 0.01, Figure 6J), *Cat* (*P* < 0.05, Figure 6K) and *Nfe2l2* (*P* = 0.052, Figure 6L), compared to the HFD-fed mice.

Taken together, these results suggest that Rb1 adjusts colonic gene expression associated with free fatty acid receptors, intestinal barrier function, and oxidative stress in HFD-fed mice, which may partly explain the mechanism of beneficial effects of Rb1.

### Rb1-Induced Changes of Gut Microbiota, Fecal Lipid Profiles, and Colonic Gene Expression Associate With Improvement of Metabolic Phenotypes in High-Fat Diet-Fed Mice

**FIGURE 7:**
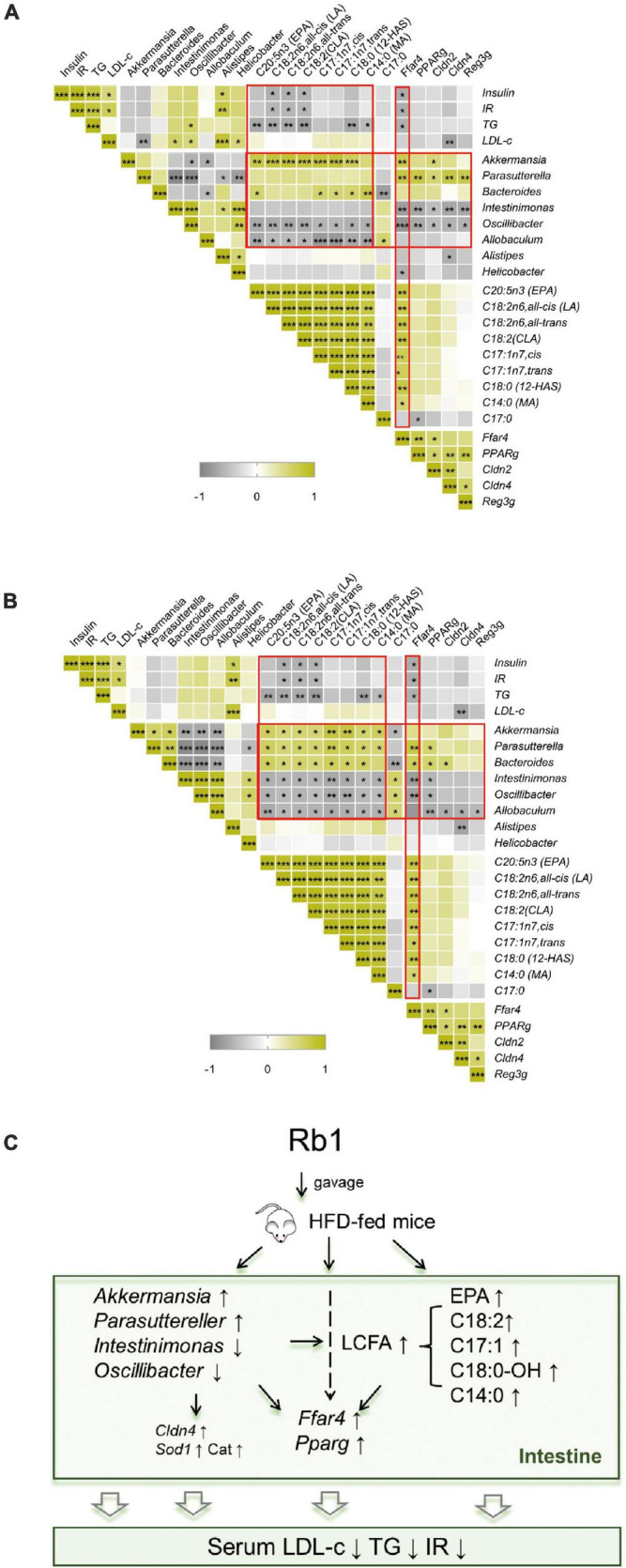
Correlation analysis and potential mechanism of improvement of metabolic disorder *via* Rb1 treatment. *Spearman* rank correlation between gut microbiota against the corresponding host metabolic parameters, fecal free fatty acids profiles or colonic gene expression for samples of day 20 **(A)** or day 27 **(B)** were individually calculated. The correlation analysis inferred potential mechanism of Rb1-treatment improved metabolic disorder is shown in panel **(C)**. *P < 0.05, **P < 0.01, ***P < 0.001.

Rb1 treatment ameliorated metabolic disorders in HFD-fed mice, accompanied by altered gut microbiota, fecal lipid profiles, and colonic gene expression. To find out whether there are correlations between physiological phenotypes and gut microbes or fecal metabolites, *Spearman* correlation analysis is used to calculate the relationship between any two of them. As shown in Figures 7A,B, Rb1 treatment decreased insulin, IR, and TG, which was positively correlated with the decreased abundance of *Alistipe* in feces, and was negatively correlated with the increased content of three octadecenoic acids (linoleic acid, linoelaidic acid, and conjugated linoleic acids) in feces, and was also negatively correlated with upregulated *Ffar4* gene expression in the colon. Additionally, eight LCFAs were increased in feces in response to Rb1 treatment, which had a positive correlation with the increased abundance of *Akkermansia* (day 20 and 27) and *Parasutterella* (day 27), but had a negative correlation with the decreased abundance of *Intestinimonas* (day 27), *Oscillibacter* (day 20 and 27) and *Allobaculum* (day 20 and 27). Moreover, the expression of fatty acid receptors genes (*Ffar4 and Pparg*) was upregulated in the colon, which was positively correlated with the increased abundance of *Akkermansia* (day 20) and *Parasutterella* (day 20 and 27), but was negatively correlated with the decreased abundance of *Intestinimonas* (day 20 and 27*)*, *Oscillibacter* (day 20 and 27) and *Allobaculum* (day 27). Similarly, increased expression of intestinal-barrier-related genes (*Cldn2*, *Cldn4, and Reg3g*) also had the same association with the trend of these microbes. Notably, the increased *Ffar4* gene expression had a significantly positive correlation with the content of eight LCFAs that were enriched by Rb1 in feces.

These results suggest that the metabolic improvement by Rb1 treatment might have a strong correlation with alteration in gut microbiota, fatty acids profiles, and colonic gene expression. In detail, Rb1 administration modulates the gut microbiota by increasing the relative abundance of *Akkermansia* and *Parasuttereller* and decreasing the relative abundance of *Intestinimonas* and *Oscillibacter*. Moreover, Rb1 administration also increases free fatty acids content in feces (mainly EPA and octadecenoic acids) and upregulates the gene expression of fatty acid receptors (*Ffar4 and Pparg*), intestinal barrier function (*Cldn4*), and oxidative stress (*Sod1* and *Cat*) in the colon, which makes beneficial effects on metabolic improvement in HFD-fed mice (Figure 7C).

## Discussion

Previous studies have demonstrated that ginsenoside Rb1 may improve metabolic disorders induced by obesity through regulating glucose and lipid metabolism in the liver and adipose tissues (Zhou et al., 2019). In this study, we found that Rb1 treatment *via* oral administration reduced serum TG, LDL-c, and insulin in HFD-induced obese mice, and the lipid-lowering effect was further verified in a hyperlipidemia rat model, which indicated that Rb1 may improve hyperlipidemia, hyperinsulinemia, and insulin resistance in metabolic disorders.

### Rb1 Treatment Modulates Gut Microbiota

Considering that ginseng-related products are mostly taken orally, the role of intestinal microbes is especially important. Due to the low bioavailability, Rb1 is enriched in the distal intestine, in which Rb1 may interact directly and thoroughly with intestinal microbes and epithelial cells, resulting in some pharmacological effects. Therefore we explored the dynamic pattern of gut microbiota in response to Rb1 treatment *via* oral administration through analyzing microbial components of fecal samples. In this study, we found Rb1 has a beneficial effect on the improvement of metabolic disorders both in mice and rats. Considering that species differences make the composition of gut bacteria significantly different, and different diets were used to set up models in mice and rats, only mice were further discussed to explore whether gut microbiota was involved in the Rb1 pharmacological effect.

The beta diversity shows that gut microbes are significantly changed within only 9 days after Rb1 treatment in HFD-fed mice. Moreover, alpha diversity was also found decreased from 9 days after Rb1 treatment, which reveals that Rb1 treatment might reduce the diversity of gut microbes. The decline in alpha diversities may be due to Rb1 induced increases in unsaturated LCFAs in the gut. Many unsaturated LCFAs have been found to have antibacterial effects and are considered to be novel antibacterial agents (Casillas-Vargas et al., 2021). The decrease in alpha diversities may also be due to upregulation of the antimicrobial-peptide-related gene *Reg3g* expression in the colon by Rb1. This finding is consistent with previous research on *Panax notoginseng* saponins (PNS) which showed that PNS decreased the diversity of the gut microbes in HFD-fed mice (Xu et al., 2020).

At the genus level, we found high-fat diet decreased the relative abundance of *Parasutterella* and *Akkermansia*, increased the relative abundance of *Intestinimonas* and *Oscillibacter*. However, the relative abundance of these genera was significantly reversed by Rb1 treatment in HFD-fed mice. ***Akkermansia*,** belonging to *Verrucomicrobia*, has been shown to represent a human gut commensal that supports host health (Cani and de Vos, 2017; Zhang et al., 2019). *Akkermansia* supplementation can reverse metabolic disorders such as hyperlipidemia and insulin resistance in diet-induced obese mice (Everard et al., 2013) and humans (Depommier et al., 2019). *Akkermansia* can improve gut barrier function (Everard et al., 2013; Li et al., 2016). A recent study found that *Akkermansia* was increased by *Panax notoginseng* saponins (PNS) in HFD-fed mice (Xu et al., 2020). Consistent with these reports, we found that *Akkermansia* was significantly increased by Rb1 treatment and was negatively associated with serum TG, insulin, and IR index. ***Parasutterella*** is defined as a core component of the human and mouse intestinal microbes (Ju et al., 2019). Clinical studies showed that *Parasutterella* was reduced in patients with NAFLD (Yun et al., 2019), gestational diabetes (Ma et al., 2020), colorectal cancer (Wang et al., 2012), and pancreatic cancer (Ren et al., 2017). However, *Parasutterella* was shown an increase in inflammatory diseases, such as irritable bowel syndrome (Chen et al., 2018). *Parasutterella* has a potential role in metabolisms of bile acid and cholesterol (Ju et al., 2019). Previous studies showed that increasing levels of *Parasutterella* correlated with improving LDL-c levels after taking resistant starch of potato in healthy adults (Bush and Alfa, 2020). Studies found that long-term treatment of *Panax ginseng* extracts can increase *Parasutterella* in rats (Sun et al., 2018). Our study found *Parasutterella* was increased in 9 days after Rb1 treatment and was negatively correlated with serum LDL-c. ***Oscillibacter*** is a potential opportunistic pathogen. It is reported to be increased in HFD-induced obese mice and was associated with increased gut permeability (Lam et al., 2012; Duca et al., 2014). *Oscillibacter* is detected to be enriched in mice with AOM/DSS-induced colorectal cancer (Wang et al., 2018). ***Intestinimonas*** has a capacity of butyrate-producing and is considered to be a beneficial genus (Kläring et al., 2013; Yang et al., 2019); however, enrichment of *Intestinimonas* is also observed in HFD-fed rats (Lin et al., 2019).

Taken together, these results demonstrate that oral administration of Rb1 modulates gut microbes, which is beneficial to improve HFD-induced metabolic disorder.

### Rb1 Treatment Increases Free Fatty Acids in Feces

As metabolites are the end products of a range of biochemical reactions, metabolic alteration may more accurately reflect the response against perturbations in organisms (Zierer et al., 2018). In this study, we studied the fecal free fatty acids profiles and found that the content of a group of LCFAs was increased in response to Rb1 treatment. EPA which is a long-chain n-3 polyunsaturated fatty acid is reported to prevent hypertriglyceridemia and maintains insulin/glucose homeostasis (Soni et al., 2019; Zhuang et al., 2020; Pal et al., 2021). LA and CLA (a type of linoleic acid isomer) have health benefits (Shen and McIntosh, 2016; Hamilton and Klett, 2021) and are associated with a lower risk of T2DM (Mousavi et al., 2021). Additionally, myristic acid (C14:0) is reported to increase desaturase activity in rat hepatocytes (Jan et al., 2004) and increase the tissue content of C20:5 n-3 and C20:3 n-6 in the rat (Rioux et al., 2005).

It was reported that a higher level of fatty acids (such as oleic acid, phosphonic acid, and lactic acid) was found in the feces of PNS-treated HFD-fed mice in the process of the improvement of metabolic disorders (Xu et al., 2020). Ginseng extract can increase *Enterococcus faecalis* which has the ability of producing myristoleic acid (C14:1) and can decrease body weight gain and the fat mass in db/db mice (Quan et al., 2020). Consistent with these results, we observed that Rb1 treatment increased EPA and octadecenoic acids in feces, which was negatively correlated with decreased insulin, IR, and serum TG in HFD-induced obese mice.

### Rb1 Treatment Upregulates Free Fatty Acid Receptor 4 Gene Expression in the Colon

Free fatty acids play important roles not only as energy sources (Hue and Taegtmeyer, 2009) but also as signaling molecules by activating specific receptors important in regulating many physiological processes (Boden, 2011; Shetty and Kumari, 2021). FFARs, activated by different chain lengths of free fatty acids, are G protein-coupled receptors (GPCRs) and are involved in the energy metabolism and immune response (Kimura et al., 2020). FFARs have been considered as targets for the treatment of metabolic disorders (Secor et al., 2021; Son et al., 2021). *Ffar4* is highly expressed in intestinal L cells in the colon (Oh et al., 2010). Activation of FFAR4 can enhance GLP-1 secretion and improve insulin resistance and chronic inflammation in obese mice (Xiong et al., 2010; Oh et al., 2014), and improve colonic permeability in inflammatory bowel diseases mice (Salaga et al., 2021). A growing number of studies report that supplementary fish oil and LCFAs, including omega-3, omega-6, can activate FFAR4 in mice and rats and bring benefits to health (Kimura et al., 2020). Studies also reported that ginsenoside Rb2, which exhibited regulatory activities in glucose and lipid metabolism, upregulated *Ffar4* expression in macrophages (Huang et al., 2017), endothelial cells, and monocytes (Sun et al., 2020). Accordingly, in this study, we found colonic gene expression of *Ffar4* was significantly upregulated by Rb1 in HFD-fed mice, which was positively correlated with fecal content of eight Rb1-increased-LCFAs, and was negatively correlated with decreased insulin, IR, and serum TG. These results suggest that LCFA increased by Rb1 might activate *Ffar4* in the intestine, which further improves insulin resistance. However, how *Ffar4* mediates insulin-sensing effects needs more experiments to explore the mechanism.

### Correlation Analysis Indicates That Gut Microbes Might Involve in Modulating Intestinal Fatty Acids Composition

Gut microbes modify host fatty acid composition by transformation, absorption, metabolism, and excretion (Lamichhane et al., 2021). In this study, we found fecal content of eight Rb1-increased-LCFAs was positively correlated with the increased abundance of *Akkermansia*, *Parasutterella*, and *Bacteroides*, and was negatively correlated with the decreased abundance of *Intestinimonas, Oscillibacter*, and *Allobaculum*, which indicates that gut microbes might involve in modulating fatty acids composition. LCFAs in the intestine mainly come from diet. Studies showed that *Oscillibacter* and *Alistipes* were positively associated with obese and diabetic phenotypes (Lam et al., 2012; Qin et al., 2012). They are enriched by a high-fat diet, indicating that they can fully utilize lipids (Lam et al., 2012; Wan et al., 2019). Therefore, the decrease of *Oscillibacter* and *Alistipes* during Rb1 treatment in HFD-fed mice may lead to reduced utilization of LCFAs. Additionally, in this study, functional pathway prediction of gut microbes shows that Rb1 treatment increases pathways including fatty acid biosynthesis and biosynthesis of unsaturated fatty acids, whereas HFD decreases these pathways, which indicates that microbes might increase fatty acids biosynthesis. Collectively, these results imply that gut microbes might participate in the regulation of intestinal fatty acids composition in HFD-fed mice.

### Limitation

The interaction between gut microbiota and the host is extremely complex. In this study, we only report the association between gut microbiota and the beneficial effect of Rb1. It needs more experiments to verify how microbes in the gut involve in the pharmacological effects of Rb1.

## Conclusion

Overall, we find Rb1 treatment by oral administration improves hyperlipidemia, hyperinsulinemia, and insulin resistance in HFD-fed mice. This metabolic improvement may benefit from the modulation of gut microbes and intestinal fatty acids profiles by Rb1 treatment. We find the relative abundance of some key bacterial genera, including *Akkermansia, Parasuttereller*, *Intestinimonas*, and *Oscillibacter* are recovered following Rb1 treatment in HFD-fed mice. Especially, the content of a group of LCFAs is significantly increased in feces in response to Rb1 treatment, and this change is notably correlated with the relative abundance of key bacterial genera in the intestine. Furthermore, this alteration of LCFAs is sensed by free fatty acid receptors in the colon, which may further lead to the improvement of energy metabolism in HFD-fed mice. This study provides an alternative mechanism for Rb1 to treat metabolic disorders induced by obesity and that may contribute to the development of new nutraceutical-based remedies accordingly.

## Data Availability Statement

The raw 16S rRNA sequencing data and metabolite abundance of each sample can be obtained through the National Omics Data Encyclopedia with the accession number OEP002877 (https://www.biosino.org/node/project/detail/OEP002877).

## Ethics Statement

Mouse experimental protocols were approved by the institutional animal care and use committee of Shanghai Institute of Nutrition and health, Chinese Academy of Sciences (Shanghai, China); Rat experimental protocols were approved by the institutional animal care and use committee of Nankai University (Tianjin, China).

## Author Contributions

GPZ gave a general research direction and manuscript revision. HZ contributed to the experimental design, data analysis, and manuscript writing. HZ, XZ, QX, LZ, and TY experimented on mice. MZ, YC, KZ, and QB experimented on rats. GYZ, HKZ, ML, SC, and YW performed bioinformatics analysis. CY gave the constructional suggestions on lipidomics analysis. QL, XY, and ZZ contributed to the research discussion. YL contributed the constructional suggestions for revision. GYZ, HKZ, CZ, and XYY contributed to manuscript revision. All authors made the final approval of the version to be submitted.

## Conflict of Interest

XZ and LZ were employed by the Zhejiang Hongguan Bio-pharma Co., Ltd., Jiaxing, China. QL was employed by the Suzhou BiomeMatch Therapeutics Co., Ltd., Shanghai, China. HZ, GPZ, and YL are inventors of patent application (202210320411.3), related to this work. The remaining authors declare that the research was conducted in the absence of any commercial or financial relationships that could be construed as a potential conflict of interest.

## Publisher’s Note

All claims expressed in this article are solely those of the authors and do not necessarily represent those of their affiliated organizations, or those of the publisher, the editors and the reviewers. Any product that may be evaluated in this article, or claim that may be made by its manufacturer, is not guaranteed or endorsed by the publisher.
