# Supplementary material for: Ginsenoside Rb1 Improves Metabolic Disorder in High-Fat Diet-Induced Obese Mice Associated With Modulation of Gut Microbiota
Source: Front Microbiol. 2022 Apr 19;13:826487. doi: 10.3389/fmicb.2022.826487 (PMC9062662; doi:10.3389/fmicb.2022.826487)

**SUPPLEMENTARY MATERIAL**

| **Supplementary Table 1 Primers for real-time quantitative** | | |
| --- | --- | --- |
| **Genes** | **Forward primer (5′ to 3′)** | **Reverse primer (5′ to 3′)** |
| Cldn2 | ATACTACCCTTTAGCCCTGACCGAGA | CAGTAGGAGCACACATAACAGCTACCAC |
| Cldn4 | ATGGCGTCTATGGGACTACAG | GAGCGCACAACTCAGGATG |
| Mu2 | CTG TAC CTA TGT GCT GGT GGA | CAT TGG CAT CAC AGT GGT AGT |
| Reg3g | TTCCTGTCCTCCATGATCAAA | CATCCACCTCTGTTGGGTTC |
| Nfe2l2 | TCTCCTAGTTCTCCGCTGCT | AAATCCATGTCCTGCTGGGA |
| Cat | AAGATTGCCTTCTCCGGGTG | GACATCAGGTCTCTGCGAGG |
| Sod1 | GTCGGCTTCTCGTCTTGCTC | CTGATGGACGTGGAACCCAT |
| Ffar1 | CCTTCGCTCTCTATGTATCTGCC | CGCAGTTTAGCGTGGGACA |
| Ffar2 | ATCCTCCTGCTTAATCTGACCC | CGCACACGATCTTTGGTAGGT |
| Ffar3 | CTTCTTTCTTGGCAATTACTGGC | CCGAAATGGTCAGGTTTAGCAA |
| Ffar4 | TCGCTGTTCAGGAACGAATG | CACCAGAGGCTAGTTAGCTG |
| Pparg | GAGAAGCTGTTGGCGGAGAT | GCTCGCAGATCAGCAGACTCT |
| Gapdh | CAGAACATCATCCCTGCATC | CTGCTTCACCACCTTCTTGA |


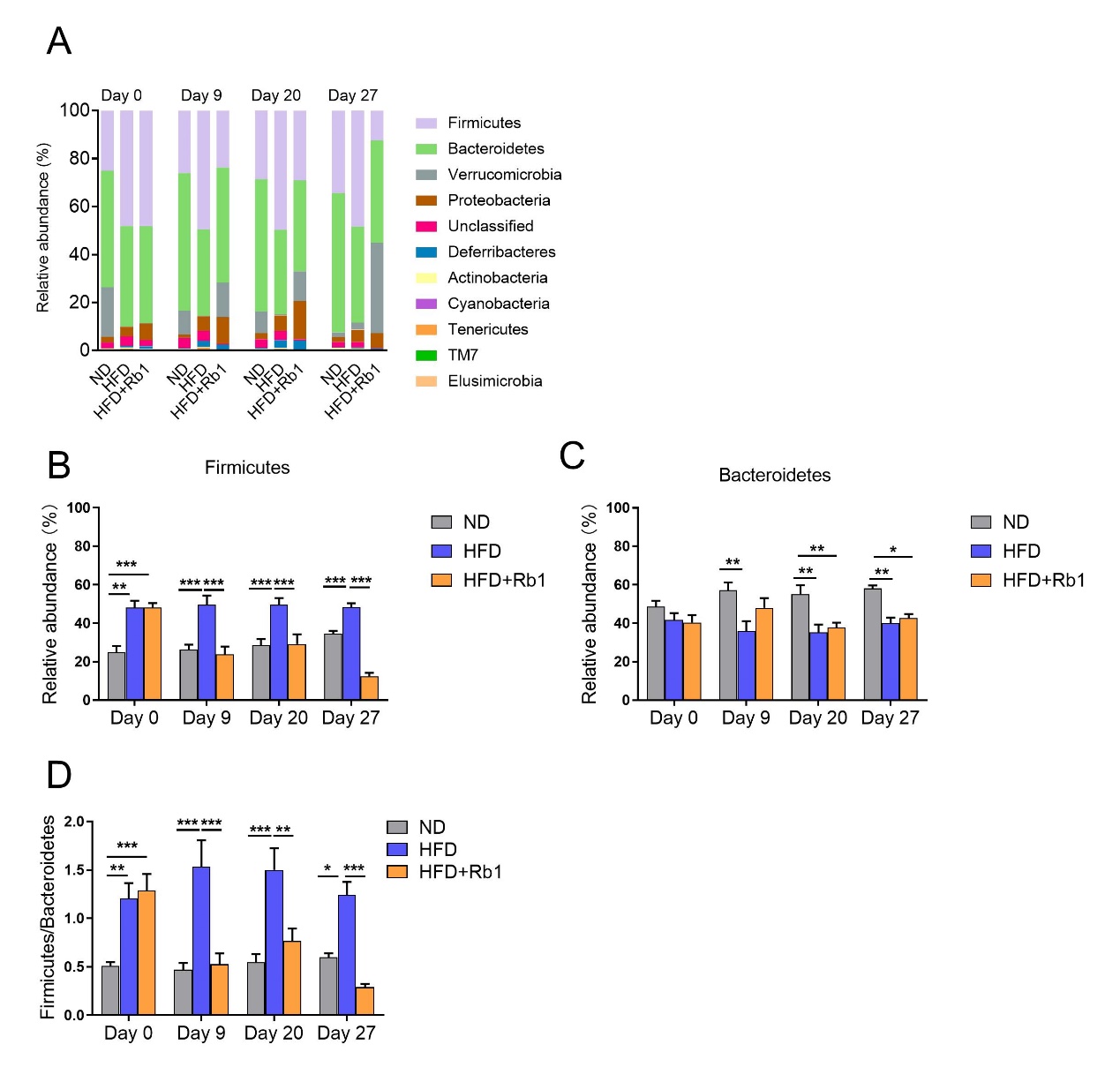


Supplementary Figure S1 Relative abundance in phylum level. **(A)** Relative abundance in phylum level. **(B)** Relative abundance of Firmicutes. **(C)** Relative abundance of Bacteroidetes. **(D)** The ratio of Firmicutes to Bacteroidetes. All data were expressed as the mean ± SEM (n=5-7 mice/group). Significant differences among groups were evaluated using a two-way analysis of variance followed by Tukey’s multiple comparison test. Abbreviations: *, *P<*0.05; **, *P<*0.01; ***, *P<*0.001. ns, not significant. ND, normal diet (ND)-fed mice; HFD, high-fat diet (HFD)-fed mice; HFD+Rb1, HFD-fed Rb1-treated mice.

Supplementary Figure S2 Linear discriminate analysis effect size (LEfSe) analysis of intestinal bacterial flora in HFD-mice. LDA scores and cladogram were generated from LEfSe results between the HFD and HFD-Rb1 groups of mice (LDA>3). **(A)** Day 9, **(B)** Day 20, **(C)** Day 27

A

B

C


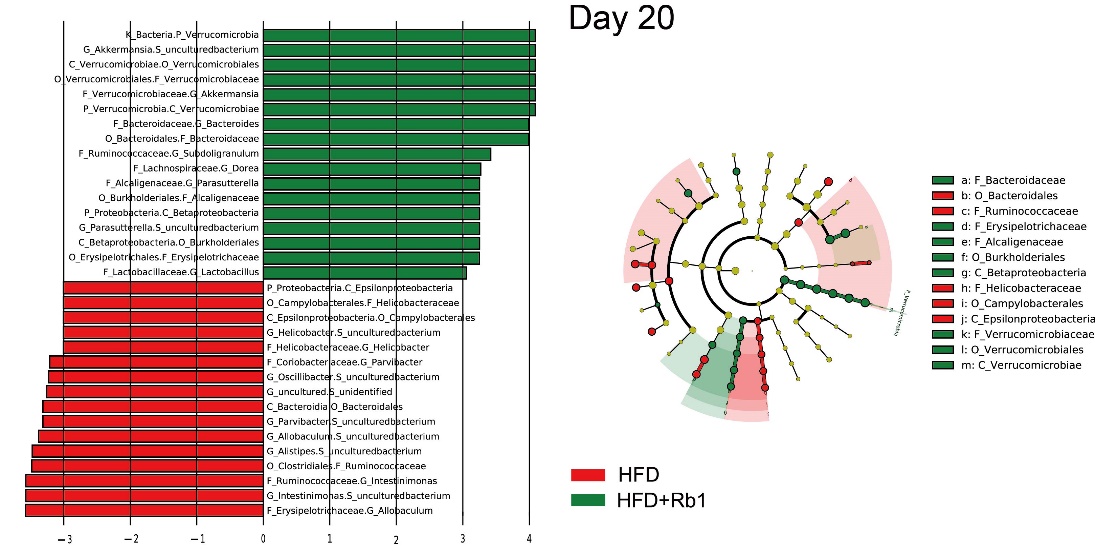

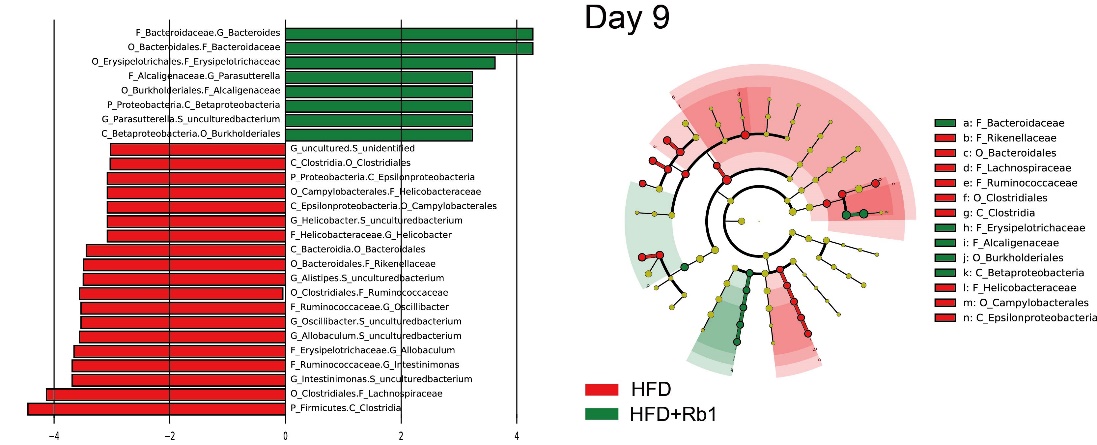

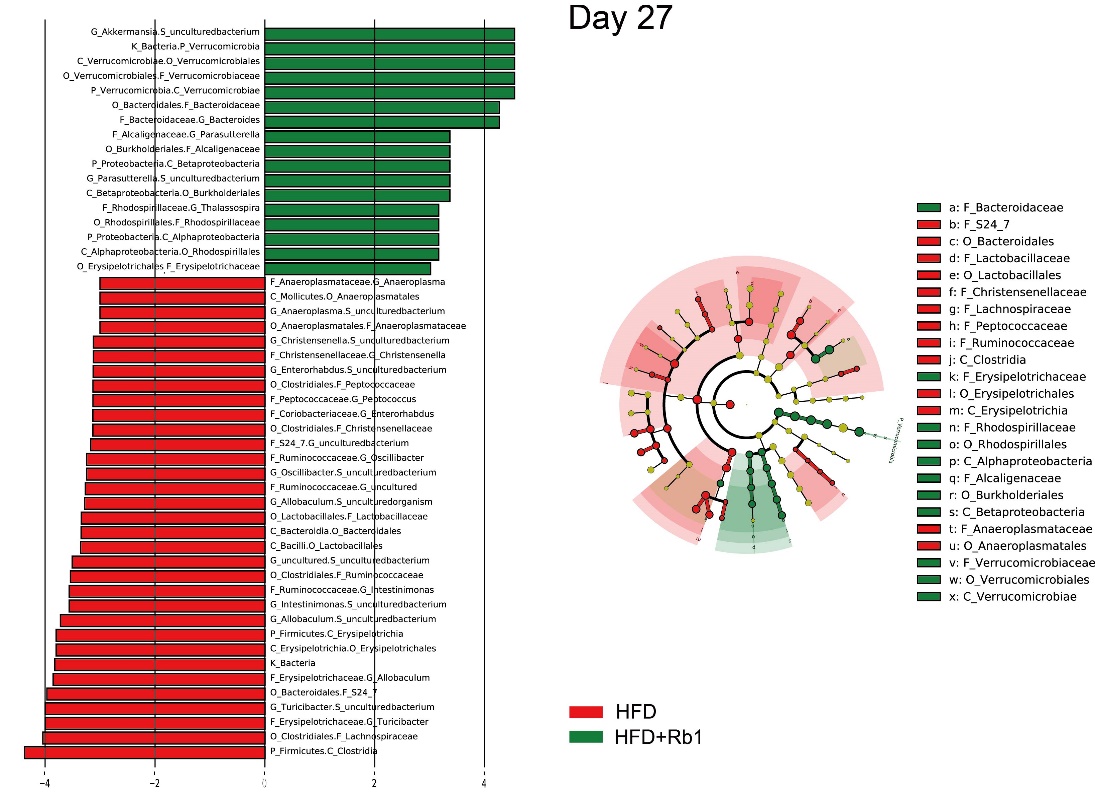

Supplement: Supplementary file 1 [file Data_Sheet_1.docx]
